# *In Vitro* Biological Screening of* Hartmannia rosea* Extracts

**DOI:** 10.1155/2017/8968604

**Published:** 2017-12-04

**Authors:** Rehana Rashid, Abida Kalsoom Khan, Ihsan Ul Haq, Sadullah Mir, Sadaf Mehmood, Yi Lu, Ghulam Murtaza

**Affiliations:** ^1^Department of Chemistry, COMSATS Institute of Information Technology, Abbottabad 22060, Pakistan; ^2^Department of Pharmacy, Quaid-i-Azam University, Islamabad 45320, Pakistan; ^3^School of Preclinical Medicine, Beijing University of Chinese Medicine, Beijing 100029, China; ^4^Department of Pharmacy, COMSATS Institute of Information Technology, Abbottabad 22060, Pakistan

## Abstract

The present study is focused on the assessment of the medicinal therapeutic potential extracts of* H. rosea* to investigate their pharmacological implications based upon science proofs. The antioxidant activity of fraction of* H. rosea*, namely, *n*-hexane (HR-1), ethyl acetate (HR-2), chloroform (HR-3), and *n*-butanol (HR-4), was performed by using the DPPH radical scavenging method. The cytotoxicity and enzyme inhibition assessment were also performed. All the extracts showed significant antioxidant, antibacterial, and protein kinase inhibition but none of the extracts exhibited *α*-amylase inhibition activity. The chloroform extract HR-3 may block a kinase receptor from binding to ATP; the lead molecule will be isolated, which may stop cancerous cell growth and demotion of cell division. It is predicted that ethyl acetate, chloroform, and *n*-butanol extracts of* H. rosea* contain polyphenolics, flavonoids, and alkaloids that are biologically effective candidates exhibiting significant cytotoxicity, antioxidant, and antimicrobial activities. They may control oxidative damage in the body tissues and act as potential antidiabetic and anticancer agents. These studies will also be helpful for future drug designing and drug development research.

## 1. Introduction

Natural products have been used for centuries for the treatment of ailments, mostly in developing countries, where the economic resources are limited and affordability and access to modern treatment is scarce [1].* Hartmannia* also known as* Oenothera* is a large and medicinally important genus of plants with approximately 124 species belonging to the Onagraceae family.* Hartmannia rosea (H. rosea)* plant species is commonly known as evening primrose, found in USA and Mexico and also widely grown in localities like Hazara, Poonch, Kashmir, and Jhelum valley in Pakistan [2].

Since herbal medicine can be used as an alternative medicine due to its cost effectiveness and lesser side effects, extensive research has been carried out to update the ethnomedicinal plant remedies through standardization and quality control for their recognition and acceptance in the international community. Numerous compounds have been isolated from genus* Hartmannia*, yet there are some therapeutically imperative species for which insufficient work is accounted.

Oil from seeds of evening primrose is a rich source of *γ*-linoleic acid, used for the production of prostaglandins and related hormones [3]. Its oral dosages have been used to treat breast disorders, atopic eczema, premenstrual tension, and rheumatoid arthritis [4]. Previously, antioxidant [5], antitumor [6], antimicrobial [7], anti-inflammatory [8], antidiarrhoeic [9], and antisecretary [10] activities of its different species have been reported.

The aqueous extracts of* Hartmannia rosea* have been used as folk medicine for headache, inflammation, cough, diarrhea, scabies, staphylococcal infection, pimples, stomatitis, liverwort, and hepatic and kidney diseases [11]. The antidiarrheal potential of decoction of* H. rosea* has been reported in the diarrhoeic mice stimulated with castor oil [9]. The aqueous and methanolic extracts of* H*.* rosea* showed significant inhibitory effect against inflammation, through the* in vivo* study done on adult female Wistar rats and male NIH mice. It has been demonstrated that these extracts did not show any perniciousness and induced significant anti-inflammatory activity [8]. The methanolic extract of aerial parts has also been used for the treatment of bruises and swelling [12]. The ethanol, acetone, and ethyl acetate fractions of* H. rosea* show antioxidative efficacy [13]. The 90% ethanolic and aqueous extracts of stem and roots of* H. rosea* exhibited anthelmintic potential [14]. The percentage inhibition of heart contractile activity of male Wistar rats was evaluated at the dose of 1.0 mg/mL [15]. The literature review interpreted that very limited research work has been accomplished on* H. rosea*, whereas most of the pharmacological potentials of this beneficial plant species are unexplored. In the present investigation antioxidant, antimicrobial, protein kinase inhibitor, *α*-amylase, and cytotoxic efficacies of different solvent extracts of* H. rosea* are reported for the first time using different protocol and strains.

## 2. Materials and Methods

### 2.1. Preparation of the* Hartmannia rosea* Extracts

The plant species* Hartmannia rosea* (Onagraceae) was collected from Abbottabad during its flowering season from April to June 2013. Methanol, *n*-hexane, ethyl acetate, chloroform, and *n*-butanol of commercial grade were purchased locally (Shalimar Chemicals, Rawalpindi, Pakistan).

The whole plant* H. rosea* was dried under shade and it was ground into powder. 5 kg of the powdered material was soaked in 10 L methanol for fortnight. The methanolic extract was filtered and dark greenish brown filtrate was evaporated on vacuum rotary evaporator (Heidolph-Hei-VAP Germany) to obtain crude extract, and this process was repeated thrice. The methanolic extract was fractionated on the basis of solvent polarity like *n*-hexane (HR-1), ethyl acetate (HR-2), chloroform (HR-3), and *n*-butanol (HR-4). The dried extracts were used for the estimation of subsequent biological activities.

### 2.2. Biological Assessments

Extracts of* H. rosea* (HR) were used for the determination of total antioxidant capacity (TAC), total reducing power (TRP) and free radical scavenging activity (RSA) by DPPH assay.

#### 2.2.1. Radical Scavenging Activity (DPPH Assay)

The antioxidant efficacy of four extracts of* H. rosea* was determined by assessing their capacity of quenching the stable 2,2-diphenyl-1-picrylhydrazyl (DPPH) free radical scavenging activity (RSA, %) by using UV spectroscopic analysis [16, 17]. The IC_50_ is the concentration at which it inhibits 50% free radical formation. 180 *μ*L of (9.2 mg/100 mL methanol) 2,2-diphenyl-1-picrylhydrazyl (DPPH) reagent was added to 96-well plates, already containing 20 *μ*L of each extract (HR-1, HR-2, HR-3, and HR-4), respectively, and each of the wells contained 200 *μ*L as final volume. Ascorbic acid and reagent were used as positive and negative controls, respectively. Then the sample well plates were incubated for 1 hour at 37°C and absorbance was taken at 517 nm by using microplate reader (Biotek-ELx800 Absorbance Reader made by Wolflabs). Experiment was performed in triplicate on each sample and radical scavenging activity (%) was calculated by using the formula

(1) $$\begin{array}{c} \text{Inhibition percentage}=Ac-\frac{As}{Ac}\times 100. \end{array}$$

As is the absorbance of the sample with reagent DPPH, while Ac is the absorbance of the control without sample. The ascorbic acid was used as standard positive control.

#### 2.2.2. Total Antioxidant Capacity (TAC)

Phosphomolybdenum method was used for the measurement of total antioxidant capacity of the extracts [18]. The extracts HR-1, HR-2, HR-3, and HR-4 were tested by mixing 0.1 mL of each extract (4 mg/mL DMSO) with 1 mL reagent (4 mM ammonium molybdate, 28 mM sodium phosphate, and 0.6 M sulphuric acid), ascorbic acid (4 mg/mL) as positive control and 1 mL reagent as blank were incubated for 90 min at 95°C. The samples were cooled down to room temperature and absorbance of each reaction mixture was measured at 695 nm (Biotek-EL x 800 Absorbance Reader made by Wolflabs). The experiment was performed in triplicate. The antioxidant capacity was expressed as the number of mg equivalent of ascorbic acid per mg of the sample. Calibration curve was prepared by taking the concentration of ascorbic acid as 125 *μ*g/mL, 100 *μ*g/mL, 75 *μ*g/mL, 50 *μ*g/mL, and 25 *μ*g/mL.

#### 2.2.3. Total Reducing Power (TRP) Estimation by Colorimetric Assay

The reducing power of four extracts was estimated by the predescribed method [19]. 100 *μ*L of each fraction, that is, HR-1, HR-2, HR-3, and HR-4 (4 mg/mL DMSO), was mixed with 400 *μ*L of 0.2 mol/L phosphate buffer of pH 6.6 and 500 *μ*L of 1% potassium ferricyanide [K_3_Fe (CN)_6_]. The reaction mixture was then incubated at 50°C for 20 min and after incubation 500 *μ*L of 10% trichloroacetic acid was added to it. Mixture was centrifuged at 3000 rpm for 10 min at room temperature. The 100 *μ*L supernatant solution was poured in 96-well plates, respectively, and 20 *μ*L distilled water was added along with 100 *μ*L ferric chloride (0.1%). DMSO with previously prescribed reaction mixture was used as blank without sample. The absorbance was measured at 630 nm on microplate reader. The reducing power was expressed as mg ascorbic acid equivalent per gram extracts.

#### 2.2.4. Cytotoxicity Measurement

*(1) Brine Shrimp Lethality Assay*. The cytotoxic efficiency of* H. rosea* extracts was tested in a 96-well plate against brine shrimp* (Artemia salina)* nauplii (Ocean 90, USA) for their lethality test as per known protocol [20]. The cytotoxicity screening of the extracts of* H. rosea* was performed by dissolving *n*-hexane (HR-1), ethyl acetate (HR-2), chloroform (HR-3), and butanol (HR-4) extracts in DMSO. The nauplii were transferred to each well plate and the test extracts were applied against* Artemia salina* for 24 hours at room temperature. The positive control doxorubicin (4 mg/mL) and negative controls DMSO were treated in similar manner. They were incubated at room temperature for 24 h. After incubation period the degree of lethality exhibited by each extract was determined through counting the number of survivors under 3x magnifying glass against illuminated background, and median lethal dose (LD_50_) of the extract fractions with >50% mortality was observed. The experiment was performed in triplicate.

### 2.3. Antimicrobial Activity Test

#### 2.3.1. Antifungal and Antibacterial Activity

Different microbes were used for testing the antimicrobial activity of* H. rosea* extracts. The following two types of assays were performed in order to determine the antimicrobial activity of four extracts. Two bacterial strains, such as* Pseudomonas aeruginosa (P. aeruginosa)* (Gram-negative) and* Staphylococcus aureus (S. aureus)* (Gram-positive), were used for the antibacterial activity. Antifungal activity was performed against* Aspergillus niger* (FCBP-0198),* Aspergillus fumigatus* (FCBP-066), and* Mucor* species (FCBP-0300).

DMSO, nutrient agar, reference antibiotic (Cefotaxime-USP) and Sabouraud dextrose agar (SDA, Merck, Germany) medium was used for antifungal assay. Dimethyl sulfoxide (DMSO) was used for the dilutions of the samples.

*Agar Disc Diffusion Method*. Agar disc diffusion method was used to check the antibacterial activity of fractions of* H. rosea* [21]. Media were prepared by dissolving 23 g of nutrient agar in 1 L of distilled water adjusting the pH of the media as 7 and autoclaved at 121°C for 20 min. 25 mL of agar medium was poured in already sterilized Petri dishes and allowed to solidify. The already prepared inoculums of each bacterium were swabbed smoothly on the surface of the media of each plate and labeled. The sterile filter paper discs of 6 mm size were impregnated with 2.5 *μ*L of fractions in DMSO by means of micropipette and kept for drying in laminar flow. The solution (1 mg/mL in DMSO) of standard antibiotic cefotaxime-USP was used as positive control and negative control blank discs were made with DMSO solvent. The test sample discs and standard and blank discs were overlaid on the surface of the agar Petri dishes. The discs were incubated at 37°C for about 24 hours. After incubation the zones of inhibition (ZOI) were measured in mm as the difference between the diameter of disc and the diameter of inhibition. The same procedure was repeated in triplicate for each sample.

Minimum inhibitory concentration (MIC) was considered as the minimal concentration of the sample fractions that inhibited the bacterial growth, after 24 h incubation at 37°C. Antifungal efficacy of the* H. rosea* fractions was estimated as triplicate analysis using the disc diffusion method [21]. Three fungal strains, namely,* Aspergillus niger* (FCBP-0198),* Aspergillus fumigatus* (FCBP-066), and* Mucor* species (FCBP-0300), were grown by using Sabouraud dextrose agar (SDA) containing 62 g/L of SDA dissolved in distilled water. The four fractions of* H. rosea*, namely, HR-1, HR-2, HR-3, and HR-4, were dissolved separately in DMSO, so that the concentration of each fraction was 20 mg/mL of DMSO. Then, sterile filter paper discs were impregnated with 5 *μ*L of each of the sample fractions. DMSO treated disc was used as negative and 20 *μ*g/disc clotrimazole (4 mg/mL) standard drug as positive control. The plates were incubated at 28°C for 24 h.

### 2.4. Inhibitory Properties against Enzymes

#### 2.4.1. Suppression of Protein Kinase

The protein kinase suppression was conducted by observing generation of hyphae (HFI) in purified isolates of* Streptomyces* 85E strain [22]. Mycelia fragments of* Streptomyces* were spread on sterile agar plates containing mineral ISP4 medium for the development of bacterial lawn. The sterile 6 mm filter paper discs were impregnated with 5 *μ*L of each fraction (20 mg/mL of DMSO) and they with final concentration of 100 *μ*g/disc were applied on the surface of* Streptomyces* 85E seeded agar plates. Surfactin standard (80-0.02% in distilled water) and DMSO infused discs were also included as positive and negative controls, respectively. All the plates with sample loaded and controls were incubated at 30°C for 72–96 h. The bald zones of inhibition around controls infused discs and samples were evaluated.

#### 2.4.2. Alpha-Amylase Inhibition Assessment

Alpha-amylase inhibition assay was performed using a modified protocol [23]. The enzyme inhibition kept the undigested starch that was determined at 630 nm (blue complex of Starch-Iodine). Phosphate buffer solution (PBS) pH 6.8 was prepared. 0.5 mg *α*-amylase (28 U/mg) containing 14.37 U was dissolved in 1 mL of phosphate buffer solution. It was then diluted to 0.12 U/mL. The stock solutions of each extract (4 mg/mL), starch 2 g/L, iodine reagent (containing 5 mM I_2_ and 5 mM potassium iodide), positive control, and acarbose (8.64 mg/mL) were prepared in DMSO. The reaction mixture contained 15 *μ*l phosphate buffer (pH 6.8), 25 *μ*l *α*-amylase enzyme, 40 *μ*l starch, and 10 *μ*l extract (100 *μ*g/mL final concentration) which were incubated for 30 min at 50°C. The reaction was terminated by adding 20 *μ*l HCl (1 M) into the reaction mixture. Then, 90 *μ*l iodine reagent was added to each well. This resulted in the change of color in those wells where starch was left due to inhibition of enzyme, and absorbance was measured at 360 nm. A blank (buffer, starch, enzyme, and DMSO), a negative control DMSO, and a positive control acarbose were used for the comparison of results. Intense blue color indicated the strong activity. The assay was performed in triplicate in 96-well microtiter plate reader. Results were expressed as percentage *α*-amylase inhibition.

(2) $$\begin{array}{c} \text{Inhibition}\left(\;\%\;\right)=\frac{\left({Ac}^{+}\right)-\left({Ac}^{-}\right)-\left(As-Ab\right)}{\left({Ac}^{+}\right)-\left({Ac}^{-}\right)}\times 100, \end{array}$$

where Ac^+^ and Ac^−^ represent positive and negative control, respectively, while As is used for sample and Ab for blank. 50% inhibition of sample and acarbose is the IC_50_ value.

### 2.5. Statistical Analysis

The results obtained for cytotoxicity and antimicrobial assays were expressed as mean ± standard error of mean (SEM). Comparisons were performed within groups by the analysis of variance using the ANOVA test. Significant differences between control and experimental groups were assessed by the Sigma Stat software. A probability level *P* < 0.05 was considered to indicate statistical significance.

## 3. Results

The extraction efficacy is not dependent only upon the change in the composition of extractable plant metabolites and the polarity of the solvents used but their biological activities are also influenced as the diversity in the nature of components has specific solubility. Hence multiple extracts of crude methanolic extract of* H. rosea* were employed for the biological evaluation.

### 3.1. Biological Assessment

#### 3.1.1. DPPH Radical Scavenging Activity of* H. rosea* Extracts (RSA, %)

The results of percent free radical scavenging activity of different extracts of* H. rosea* demonstrated that ethyl acetate (HR-2) extract (IC_50_ 7.37 *μ*g/mL) and *n*-butanol extracts (HR-4) (IC_50_  7.60 *μ*g/mL) showed highest antioxidant activity, while chloroform extract (HR-3) with IC_50_ 11.40 and *n*-hexane (HR-1) have the lowest (IC_50_  15.30 *μ*g/mL) activity equivalent to standard ascorbic acid at 50 *μ*g/mL = 91.75% and IC_50_ 25.38 *μ*g/mL (Table 1).

#### 3.1.2. Total Antioxidant Capacity

Total antioxidant capacity (TAC) of various extracts of* H. rosea* is studied. The Mo(VI) has been reduced by the antioxidant extract to Mo(V) and the absorption of resultant phosphate/Mo(V) complex of green color was measured at 695 nm [24].

**Figure 1:**
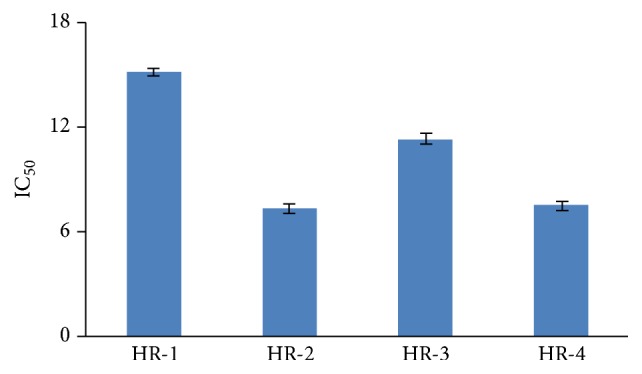
Total antioxidant capacity (TAC) mg/mL in different solvent extracts of* H. rosea*. Values are presented as mean ± standard error from triplicate experiment. HR-1: *n*-hexane, HR-2 ethyl acetate, HR-3: chloroform, and HR-4: *n*-butanol.

The ethyl acetate fraction (HR-2) and *n*-butanol extract (HR-4) showed more significant results at the ascorbic acid equivalent *μ*g/mg sample of 419.71 and 328.13, respectively, while other extracts were less effective (Figure 1).

#### 3.1.3. Total Reducing Power (TRP)

**Figure 2:**
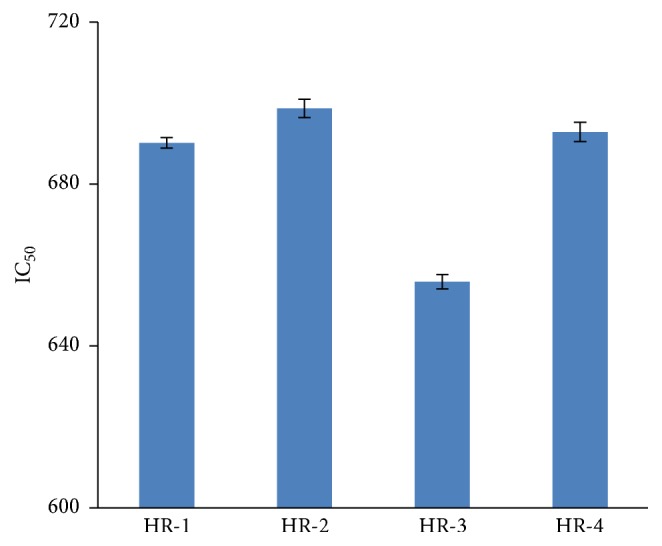
Total reducing power (TRP) mg/mL determination in different solvent extracts of* H. rosea* values is presented as mean ± standard error from triplicate experimentation.

The reducing power of *n*-hexane (HR-1), ethyl acetate (HR-2), chloroform (HR-3), and *n*-butanol (HR-4) extracts (100 *μ*L concentration) is exhibited (Figure 2). Hence ethyl acetate (HR-2) 698.7 *μ*g/mg and *n*-butanol extract (HR-4) 692 *μ*g/mg were found more active than other extracts, since the extracts having the reducing power in fact have the power of electron donation and are capable of reducing the oxidized intermediates formed in lipid peroxidation phenomena [25].

### 3.2. Cytotoxicity of* H. rosea* Extracts

The brine shrimp lethality assay is suitable and simple too for primary assessment of toxicity. The median lethal dose of the toxicity (LD_50_) for the* H. rosea* extracts HR-1, HR-2, HR-3, and HR-4 is presented in Table 2. The extracts of* H. rosea* were screened for cytotoxic activity against brine shrimp larvae. The extracts resulting LD_50_ value was determined at different concentrations, 200, 100, 50, and 25 *μ*g/mL. Among all the extracts of* H. rosea* the ethyl acetate extract (HR-2) and chloroform extract (HR-3) having LD_50_ > 20 were considered significantly active and these extracts have the potential to be the candidates for the investigation of cytotoxic compounds in them (Table 2).

### 3.3. Antimicrobial Activity of* H. rosea* Extracts

#### 3.3.1. Antifungal Activity

The antifungal potential of different solvent extracts of* H. rosea* was evaluated for the first time against three fungal strains named* Aspergillus niger* (FCBP-0198),* Aspergillus flavus* (BP-0064), and* Fusarium solani*. The antifungal growth inhibition results are presented in Table 3.

It indicated that *n*-butanol (HR-4) showed antifungal activity with 8.73 mm of the zone of inhibition against* A. flavus*. Chloroform (HR-3) and *n*-hexane (HR-1) extracts showed antifungal activity with the zone of inhibition at 9.70 mm and 8.57 mm, respectively, against* A. flavus* and 8.22 mm and 6.75 mm against* F. solani*, while *n*-butanol extracts remain inactive against both fungal strains. Ethyl acetate (HR-2) showed significant antifungal activity against* A. niger* fungal strain and* A. flavus* with the zone of inhibition of 11.82 and 11.75 mm. These most significant extracts would lead to the isolation of active constituents.

#### 3.3.2. Antibacterial Activity

Antibacterial activity of* H. rosea* extracts was performed on two bacterial strains named* Pseudomonas aeruginosa* and* Staphylococcus aureus* (ATCC-6538). The data obtained exhibited that ethyl acetate (HR-2) showed significant antibacterial activity against both bacterial strains with percentage inhibition at 21.74 *μ*g/mL and 40.58 *μ*g/mL, respectively.

However chloroform fraction (HR-3) showed minimum inhibition concentration (MIC) at 100 *μ*g/mL and percentage inhibition 47.21 against* P. aeruginosa* bacterial strain, whereas *n*-butanol (HR-4) extract showed remarkable activity on* S. aureus* bacterial strain with percentage inhibition 44.72 *μ*g/mL. Chloroform and *n*-butanol extracts exhibited significantly (*P* < 0.05) higher antibacterial activity as compared to standard drug cefixime (Table 4).

### 3.4. Enzymatic Activities of* Hartmannia rosea* Extracts

#### 3.4.1. Alpha-Amylase Assay

Type II diabetes can be managed by inhibiting hydrolyses of the carbohydrates and retaining the rate of D-glucose absorption into blood. Mean IC_50_ values of the *α*-amylase investigated for extracts of* H. rosea* did not show any significance, that is, IC_50_ > 200 *μ*g/mL, so none of the sample fractions was active, since *α*-amylase inhibition for standard acarbose is 250 *μ*g/mL with percentage inhibition 65.56% (Table 5). *α*-Amylase inhibitors as lead molecules in the extract may have different mode of inhibitory action and may be more suitable for the treatment of diabetes.

#### 3.4.2. Protein Kinase Inhibition Assay

The mechanism involved in the protein kinase activity is the transfer of phosphate group from ATP molecule and attached covalently to the serine, threonine, or tyrosine residues of specific protein and produces ADP as a byproduct. Protein kinase activity test was performed on different extracts of* H. rosea* for the first time. Only the chloroform extract (HR-3) gave remarkable clear zone of inhibition 22 mm at 100 *μ*g/disc, and 11 mm bald phenotype was formed around HR-3 extract as compared to other tested extracts. It might block a kinase receptor from binding to ATP, preventing the phosphorylation that would benefit the cancerous cell and promote cell division. The results are presented in Table 6.

The nontoxic effect of negative control (DMSO) was proved on account of lacking in inhibition zone growth. The positive control surfactin established a pronounced bald growth inhibition zone.

## 4. Discussion

There has always been search for the new constituents from herbs due to several limitations of modern drugs, since plant extracts contain tremendously active small biomolecules with selective activities in natural proportion that can contribute and exhibit acute effects in various conditions such as an oxidative stress, inflammation, and diabetes through different mechanisms.

The multiple solvent extracts of* H. rosea* were evaluated for their antioxidant potential by using DPPH as a free radical source upon reduction by accepting the hydrogen radical or electron from the sample donor antioxidant and decolorizing methanolic solution of DPPH to a yellow colored solution. All the extracts exhibited significant (*P* < 0.05) DPPH scavenging activity, in particular ethyl acetate extract (Table 1). These results indicated that polyphenols and flavonoid constituents are major contributors of the extracts, so their pharmacological efficacies could be attributed to the presence of such valuable principle molecules including quercetin 3-O-*β*-dallopyranoside-3′′,6′′-diacetate (1) and two known compounds, ursolic acid (2) and gallic acid [26].

Total antioxidant capacity (TAC) of ethyl acetate (HR-2) and *n*-butanol extracts (HR-4) showed more significant results at the ascorbic acid equivalent *μ*g/mg sample (Table 2). Total reducing power (TRP), in the extracts of* H. rosea*, has been reported for the first time. The reducing power is normally associated with the reductones that have been correlated with antioxidant capability of plant extracts [26, 27] (Figure 2).

The antioxidant activity also revealed that moderately best polar solvent extraction would be recommended for the isolation of constituents with cytotoxicity to be used as anticancer and antiobesity in adipogenesis [26] (Table 2).

Microscopic organisms responsible for fungal infections are capable of attacking the epithelial tissues so antifungal agents are used to inhibit the fungi growth or to kill the fungi. These agents act against fungal infection by three different modes, that is, inhibition of cell division, inhibition of cell wall formation, and cell membrane disruption. The antifungal potential of different solvent extracts of* H. rosea* was evaluated for the first time against three fungal strains named* Aspergillus niger* (FCBP-0198),* Aspergillus flavus* (BP-0064), and* Fusarium solani*. The antifungal growth inhibition results are presented in Table 3. Antibacterial agents are used to treat the infections caused by bacteria. They have the ability to fight against bacterial cells instead of animal cells because of the difference in the biosynthetic processes and structure of the bacterial and animal cells. The antibacterial agents act against bacterial cells through three different mechanisms. The first method is the inhibition of cell metabolism of microorganism by resisting an enzyme catalyzed reaction that occurs only in bacterial cell and not in animal cell. Second method is the inhibition of bacterial cell wall that ultimately results in the cell death and cell lyses. Third method includes the interaction of antibacterial agents with the plasma membrane of bacterial cells that affects the permeability of membrane. Conclusively, *n*-butanol extracts of* H. rosea* have the most significant antibacterial activity among all extracts (Table 4).

*α*-Amylase is a protein enzyme present in human secretions, fungi, and seeds containing starch [28]. It is responsible for the hydrolyses of polysaccharides such as carbohydrates, glycogen, and starch, which result in the formation of maltose, dextrin, glucose, and glucoamylase. It serves as the major digestive enzyme and helps in intestinal absorption [29]. Since *α*-amylase is responsible for the breakdown of carbohydrates long chain, diabetes mellitus disease characterized by hyperglycemia can be controlled through inhibition of *α*-amylase. Particularly, type II diabetes can be managed by inhibiting hydrolyses of the carbohydrates and retaining the rate of D-glucose absorption into blood. Since *α*-amylase inhibition for standard acarbose is 250 *μ*g/mL with percentage inhibition 65.56%, *α*-amylase inhibitors as lead molecules in the extract may have different mode of inhibitory action and may be more suitable for the treatment of diabetes.

As the protein kinase targeted therapeutics in cancer is promising, though it is not specified and may not be used in the treatment, however, such mioties are required to comprehend physiological roles of protein kinase [30]. The mechanism involved in the protein kinase activity is the transfer of phosphate group from ATP molecule attached covalently to the serine, threonine, or tyrosine residues of specific protein and produces ADP as a byproduct [31, 32]. To comprehend the efficacy of the HR extracts as anticancer agent, protein kinase activity test was performed on different extracts of* H. rosea* for the first time. Protein kinase activity is a promising anticancer feature in drug designing; thus HR-3 extract will be further studied for the isolation of active constituents and studying their mechanism of action.

## 5. Conclusion

*Hartmannia rosea* has a tradition of worldwide potential use as medicinal plant. It is concluded from the present studies that ethyl acetate, chloroform, and *n*-butanol extracts of this plant are biologically most effective. Therefore they will be the prospecting study targets for the discovery of lead principle constituents and their pharmaceutical potential and drug designing.

## Figures and Tables

**Table 1:** IC_50_ values for DPPH radical scavenging activity of *Hartmannia rosea* extracts. Values are presented as the mean ± SEM of experiments performed in triplicate.

Plant extract	IC_50_ Value	RSD (%)
*n*-Hexane (HR-1)	15.30 ± 0.20^*∗*^	1.315
Ethyl acetate (HR-2)	7.37 ± 0.26^*∗*^	3.575
Chloroform (HR-3)	11.40 ± 0.30^*∗*^	2.631
*n*-Butanol (HR-4)	7.60 ± 0.26^*∗*^	3.481
Ascorbic acid (As)	25.38	91.75

*Note*. *∗* indicates significant results (*P* < 0.05).

**Table 2:** Median lethal dosage (LD_50_) of *H. rosea* extracts against brine shrimp lethality.

Plant extracts	Percent mortality				LD_50_
	200 (*µ*g/mL)	100 (*µ*g/mL)	50 (*µ*g/mL)	25 (*µ*g/mL)	(*µ*g/mL)
*n*-Hexane (HR-1)	100	100	100	100	<25
Ethyl acetate (HR-2)	20	10	10	0	>200
Chloroform (HR-3)	50	10	10	0	200.4
*n*-Butanol (HR-4)	100	100	100	100	<25

**Table 3:** Antifungal activity of different solvent extracts of *H. rosea*.

Plant extracts	Zone of inhibition (mm)		
	*Aspergillus niger*	*Aspergillus flavus*	*Fusarium solani*
*n*-Hexane (HR-1)	—	8.57^*∗*^	6.75^*∗*^
Ethyl acetate (HR-2)	11.82^*∗*^	11.75^*∗*^	7.68^*∗*^
Chloroform (HR-3)	—	9.70^*∗*^	8.22^*∗*^
*n*-Butanol (HR-4)	—	8.73^*∗*^	—

*Note*. *∗* indicates significant results (*P* < 0.05) (— means not active). All the extracts exhibited significantly (*P* < 0.05) lower antifungal activity than that of standard, clotrimazole. However, ethyl acetate extract of *H. rosea* showed the highest antifungal activity among all extracts.

**Table 4:** Antibacterial activity of different solvent extracts of *H. rosea*.

Plant extracts	*Pseudomonas aeruginosa*		*Staphylococcus aureus*	
	Percentage inhibition at 100 *µ*g/mL	MIC	Percentage inhibition at 100 *µ*g/mL	MIC
*n*-Hexane (HR-1)	9.63	>100	8.67^*∗*^	>100
Ethyl acetate (HR-2)	10.68	>100	21.74	>100
Chloroform (HR-3)	47.21	100.0	39.67^*∗*^	>100
*n*-Butanol (HR-4)	17.68	>100	44.72^*∗*^	>100

*Note*. *∗* indicates significant results (*P* < 0.05). Chloroform and *n*-butanol extracts exhibited significantly (*P* < 0.05) higher antibacterial activity than that of standard, cefixime. In addition, *n*-butanol fraction *H. rosea* extract showed the highest antibacterial activity among all extracts.

**Table 5:** Alpha-amylase inhibition activity of different solvent extracts of *H. rosea*.

*α*-Amylase inhibition (%) at 200 *µ*g/mL	
Plant extracts	IC_50_ (*µ*g/mL)
*n*-Hexane (HR-1)	>200
Ethyl acetate (HR-2)	>200
Chloroform (HR-3)	>200
*n*-Butanol (HR-4)	>200

**Table 6:** Protein kinase inhibition of different solvent extracts of *H. rosea*.

Protein kinase inhibition activity	
Plant extracts	Zone of inhibition in mm at 100 *µ*g/disc
*n*-Hexane (HR-1)	—
Ethyl acetate (HR-2)	—
Chloroform (HR-3)	22.0^*∗*^
*n*-Butanol (HR-4)	—

*Note*. *∗* indicates significant results (*P* < 0.05). The chloroform extract exhibited significantly (*P* < 0.05) higher protein kinase activity compared to surfactin standard.
